# *MAP3K7* is recurrently deleted in pediatric T-lymphoblastic leukemia and affects cell proliferation independently of NF-κB

**DOI:** 10.1186/s12885-018-4525-0

**Published:** 2018-06-18

**Authors:** David M. Cordas dos Santos, Juliane Eilers, Alfonso Sosa Vizcaino, Elena Orlova, Martin Zimmermann, Martin Stanulla, Martin Schrappe, Kathleen Börner, Dirk Grimm, Martina U. Muckenthaler, Andreas E. Kulozik, Joachim B. Kunz

**Affiliations:** 1 Department of Pediatric Oncology, Hematology, Immunology and Pulmonology, Heidelberg University Children’s Hospital, Heidelberg, Germany; 2 Molecular Medicine Partnership Unit (MMPU), Heidelberg, Germany; 3 German Cancer Consortium (DKTK), Heidelberg, Germany; 4 Department of Pediatric Hematology and Oncology, MH Hannover, Hannover, Germany; 5 Department of Pediatrics, University Medical Center Schleswig-Holstein, Campus Kiel, Kiel, Germany; 6 Department of Infectious Diseases, Virology, Heidelberg University Hospital, Heidelberg, Germany; 7 German Center for Infection Research (DZIF), Partner Site Heidelberg, Heidelberg, Germany; 8 BioQuant Center, Heidelberg University, Heidelberg, Germany; 9 Cluster of Excellence CellNetworks, Heidelberg University, Heidelberg, Germany

**Keywords:** T-cell acute lymphoblastic leukemia, T-ALL, TGF-beta activated kinase 1, *MAP3K7*, chr6q15 deletion

## Abstract

**Background:**

Deletions of 6q15–16.1 are recurrently found in pediatric T-cell acute lymphoblastic leukemia (T-ALL). This chromosomal region includes the mitogen-activated protein kinase kinase kinase 7 (*MAP3K7*) gene which has a crucial role in innate immune signaling and was observed to be functionally and prognostically relevant in different cancer entities. Therefore, we correlated the presence of *MAP3K7* deletions with clinical parameters in a cohort of 327 pediatric T-ALL patients and investigated the function of *MAP3K7* in the T-ALL cell lines CCRF-CEM, Jurkat and MOLT-4.

**Methods:**

*MAP3K7* deletions were detected by multiplex ligation-dependent probe amplification (MLPA). T-ALL cell lines were transduced with adeno-associated virus (AAV) vectors expressing anti-*MAP3K7* shRNA or a non-silencing shRNA together with a GFP reporter. Transduction efficiency was measured by flow cytometry and depletion efficiency by RT-PCR and Western blots. Induction of apoptosis was measured by flow cytometry after staining with PE-conjugated Annexin V. In order to assess the contribution of NF-κB signaling to the effects of *MAP3K7* depletion, cells were treated with TNF-α and cell lysates analyzed for components of the NF-κB pathway by Western blotting and for expression of the NF-κB target genes *BCL2*, *CMYC*, *FAS*, *PTEN* and *TNF-*α by RT-PCR.

**Results:**

*MAP3K7* is deleted in approximately 10% and point-mutated in approximately 1% of children with T-ALL. In 32 of 33 leukemias the deletion of *MAP3K7* also included the adjacent *CASP8AP2* gene. *MAP3K7* deletions were associated with the occurrence of *SIL-TAL1* fusions and a mature immunophenotype, but not with response to treatment and outcome. Depletion of *MAP3K7* expression in T-ALL cell lines by shRNAs slowed down proliferation and induced apoptosis, but neither changed protein levels of components of NF-κB signaling nor NF-κB target gene expression after stimulation with TNF*-*α.

**Conclusions:**

This study revealed that the recurrent deletion of *MAP3K7/CASP8AP2* is associated with *SIL-TAL1* fusions and a mature immunophenotype, but not with response to treatment and risk of relapse. Homozygous deletions of *MAP3K7* were not observed, and efficient depletion of *MAP3K7* interfered with viability of T-ALL cells, indicating that a residual expression of *MAP3K7* is indispensable for T-lymphoblasts.

**Electronic supplementary material:**

The online version of this article (10.1186/s12885-018-4525-0) contains supplementary material, which is available to authorized users.

## Background

**Fig. 1:**
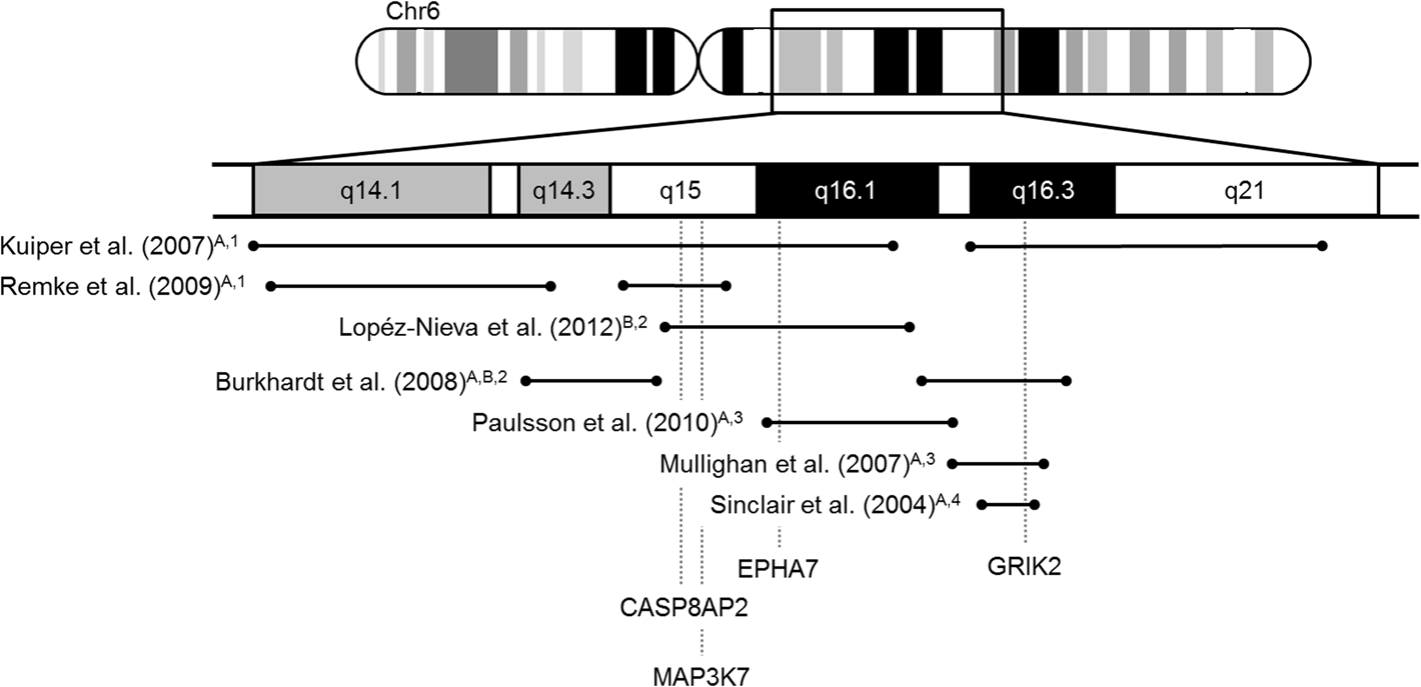
Regions of minimal deletion (RMD) on chr6q in pediatric acute lymphoblastic leukemia (ALL) and/or T-cell lymphoblastic lymphoma (T-LBL) as identified in published studies since 2004. The RMD results were derived from array comparative genomic hybridization (CGH)^1^, loss of heterozygosity analysis (LOH)^2^, single nucleotide polymorphism array analysis (SNP array)^3^ and fluorescence in situ hybridization (FISH)^4^. Originally described nucleotide positions of the proximal and distal boundaries of each RMD were adjusted to the current reference genome GRCh38/hg38 (released in 12/2013). Relative positions of previously suggested potential tumor suppressor genes (EPHA7, GRIK2) and *CASP8AP2/MAP3K7* are shown by dashed lines. ^A^ RMD derived from studies of pediatric B- and/or T-ALL samples. ^B^ RMD derived from studies in T-LBL

T-cell acute lymphoblastic leukemia (T-ALL) is an aggressive malignancy of thymocyte progenitors and constitutes 10–15% of pediatric ALL [1]. With current treatment protocols, approximately 80% of children suffering from T-ALL are cured [2, 3]. In a series of 73 primary T-ALL patient samples, Remke et al. (2009) identified recurrent deletions of 6q14.1–14.3 and 6q15–16.1 that were associated with an unfavorable response to treatment [4]. Of the 16 genes localized in these regions, the mRNA expression of *CASP8AP2* has been shown to be most strongly affected by the deletion and *CASP8AP2* has been suggested to be a tumor suppressor [4]. Similar 6q15 deletions were found in several cohorts of childhood ALL and T-cell lymphoblastic lymphomas (T-LBL) [5–10] as well as in other hematological malignancies [11–14] and in solid tumors like breast, gastric and prostate cancer [15–18] (Fig. 1). By whole exome sequencing of T-ALL samples, we had found recurrent (2/13) mutations in the *MAP3K7* gene which is included within the commonly deleted region 6q15–16.1 [19].

*MAP3K7* codes for a mitogen-activated protein kinase that has alternatively been termed *TAK1* (transforming growth factor beta activated kinase 1) [20]. It is involved in various inflammatory and immune signaling pathways like T-cell receptor, Toll-like receptor and TNF-α signaling [21]. Several stimuli lead to association with TAK1-binding proteins (TAB1–3) [22] followed by an activation of other mitogen-activated protein kinases (ERK, JNK, p38) and the transcription factor NF-κB [23]. Consistently, *MAP3K7* knockout in mice resulted in NF-κB inactivation in T-cells [24] and led to the development of myelomonocytic leukemia [25]. However, its distinct biological functions and relevance for different tumor entities appear to be cell type-specific and remain controversial [26]. For instance, in prostate cancer *MAP3K7* deletion is associated with an advanced tumor stage, lymph node metastasis and an early biochemical recurrence [16, 27], and suppression of *MAP3K7* has been shown to promote tumorigenesis [28]. In contrast, inhibition of MAP3K7 in breast cancer cells reduced tumor growth and impaired metastasis [29–31]. In an AML xenograft model, inhibition of MAP3K7 attenuated leukemia development [32].

In order to investigate the clinical relevance of deletions of *MAP3K7* in pediatric T-ALL, we analyzed a cohort of 327 primary T-ALL patient samples for *MAP3K7* deletions and correlated *MAP3K7* status with clinical features. The functional relevance of reduced *MAP3K7* expression in T-ALL cell lines was investigated by analyzing the effects of shRNA-mediated *MAP3K7* depletion on cell proliferation, apoptosis and NF-κB activation.

## Methods

### Patients

Patients were treated according to ALL-BFM 2000 [3] or AIEOP-BFM ALL 2009 protocols. These trials were registered at www.clinicaltrials.gov (#NCT00430118 and #NCT01117441). The institutional review boards of Hannover Medical School (Nr. 2522, November 9th, 2000 and December 22nd, 2008) and University of Schleswig-Holstein (A 177/09, March 12th, 2010) approved the trials. Informed consent was obtained in accordance with the declaration of Helsinki. For further details refer to the Additional file 1: Supplemental Methods.

### MLPA

Multiplex ligation-dependent probe amplification (MLPA) was performed using the MRC Holland (Amsterdam, The Netherlands) SALSA MLPA probe mix P383-A1 TALL with three additional probes for the *MAP3K7* gene according to the manufacturer’s instructions. The *MAP3K7* probe sequences were:

- Exon 1: 5´ GGGTTCCCTAAGGGTTGGACATGTCTACAGCCTCTGCCGCCTCCTCCTCCTCCTCGTCTTC/ GGCCGGTGAGATGATCGAAGCCCCTTCCCAGGTCCTCAACTCTAGATTGGATCTTGCTGGCAC 3´
- Exon 5: 5´ GGGTTCCCTAAGGGTTGGACCACGCAATGAGTTGGTGTTTACAGTG/ TTCCCAAGGAGTGGCTTATCTTCACAGCATGCAACCCAAAGCGTCTAGATTGGATCTTGCTGGCAC 3´
- Exon 7: 5´ GGGTTCCCTAAGGGTTGGACGTCTTCAGCTGGGGTATTATTCTTTGGGAAGTGATAACGCG/ TCGGAAACCCTTTGATGAGATTGGTGGCCCAGCTTCTAGATTGGATCTTGCTGGCAC 3´

Polymerase chain reaction (PCR) products were separated by capillary electrophoresis on an ABI-3130XL device. The size standard was GeneScan 500–250 (both Applied Biosystems).

### Cell culture

HEK293T cells were cultured in DMEM medium and CCRF-CEM, MOLT-4 and Jurkat cells in RPMI 1640 medium (both Gibco). Media were supplemented with 10% fetal bovine serum and 100 μg/mL penicillin-streptomycin (Biochrom). All cell lines were cultured at 37 °C and 5% CO_2_.

### AAV vector production

AAV vectors were produced as described previously [33, 34]. Briefly, HEK293T cells were triple-transfected with equal amounts of an AAV helper plasmid (encoding AAV *rep* and *cap* genes), an AAV vector plasmid encoding the anti-MAP3K7 shRNA or a non-silencing shRNA and an adenoviral helper construct. The *cap* gene AAVrh10A2 was used for the CCRF-CEM cell line and AAV9A2 for the Jurkat and MOLT-4 cell lines. These are synthetic AAV *cap* genes that were created through insertion of short re-targeting peptides into wild-type AAVrh10 or AAV9, as recently described [35]. Sense strand nucleotide sequences for the three anti-MAP3K7 shRNAs were (positions according to hg38):

- shRNA 1 5’ GTGTGTCTTGTGATGGAATA 3′, chr 6:90,561,667–90,561,622
- shRNA 2 5’ GCAAGTTCCTGCCACAAATGA 3′, chr 6:90,548,099–90,548,119
- shRNA 3 5’ GGACATTGCTTCTACAAATAC 3′, chr 6:90,548,147–90,548,167.

Sense strand nucleotide sequence of non-silencing control shRNA:

- shRNA ns 5’ GTAACGACGCGACGACGTAA 3’

See Additional file 1: Supplemental Methods for further details on shRNA design and cloning, as well as on AAV vector production.

### Transduction of T-ALL cell lines

T-ALL cells were seeded at a density of 40 cells/μl in 12-well plates and transduced with the shRNA-encoding AAV vectors at a multiplicity of infection (MOI, i.e., vector genomes per cell) between 1*10^4^ to 5*10^5^. Cells were incubated for 72 h, spun down and resuspended in fresh medium. For further experiments, cells were either re-seeded at a density of 40 cells/μl for proliferation analysis or fixed by adding paraformaldehyde (PFA) to a final concentration of 4% in phosphate-buffered saline (PBS, Sigma) and incubating for 30 min at room temperature. After washing with PBS, transduction rates were measured by detection of the green fluorescent protein (GFP) reporter that is co-encoded by all AAV/shRNA vectors by means of flow cytometry (Cytomics FC500 MPL analyzer, Beckman Coulter).

### Annexin V cell death assay

Six days after transduction, T-ALL cells were counted and pelleted for staining with an Annexin V-R-Phycoerythrin (PE) conjugate (BD Biosciences). Cells were washed twice in Annexin V binding buffer and then incubated with Annexin V-PE for 15 min at room temperature. Next, they were fixed with 4% PFA/PBS, washed once with PBS, and analyzed by flow cytometry. See Additional file 1: Figure S3 for FACS dot plots and Additional file 1: Supplemental Methods for further description.

### Quantitative real-time-PCR (qRT-PCR)

Six days after transduction, total RNA was extracted by using the RNeasy Mini kit (Qiagen) in accordance with the manufacturer’s instructions. cDNA was synthesized by using oligo(dT) primers and RevertAid H Minus M-MuLV Reverse Transcriptase (Thermo Scientific) according to the manufacturer’s instructions. All quantitative RT-PCR reactions were performed in triplicates by an ABI StepOnePlus thermocycler (Applied Biosystems) with SYBR Green PCR Master Mix (Thermo Scientific). Primer sequences are listed in Additional file 1: Table S1.

### Western blotting

To obtain whole cell lysates, cells were extracted six days after transduction by use of Mammalian Protein Extraction Reagent (M-PER, Thermo Fisher Scientific) and subjected to at least three freeze-thaw cycles (− 80 °C/room temperature). Samples were run on a sodium-dodecyl sulfate/ 10% polyacrylamide gel and blotted onto a Westran S polyvinylidene difluoride membrane (GE Health Care). After blocking for 24 h in a 5% milk solution, proteins of interest were detected by incubation for 24 h with antibodies against MAP3K7, NF-κB p100/p52, NF-κB p105/p50, IκB (all Cell Signaling) used at a 1:1000 dilution in 5% milk/TBS-T (Tris-buffered saline with 0.5% Tween-20) or 5% BSA (bovine serum albumin)/TBS-T for phospho NF-κB p65 Ser536 (Cell Signaling). Incubation for 1 h with a peroxidase-conjugated anti-mouse or anti-rabbit antibody (both Sigma) was followed by signal detection with Western Lightning Plus-ECL detection reagent (PerkinElmer) using the Fusion FX detection system (Vilber Lourmat). ImageJ (1.48v) was used for quantification of results.

### Statistical analysis and graph preparation

Event-free survival (EFS) was defined as the time from diagnosis to the date of last follow-up in complete remission or first event. Events were non-response (defined as not achieving a complete remission after the first HR-block in ALL-BFM 2000 and after the third HR-block in AIEOP-BFM ALL 2009), relapse, secondary neoplasm, or death from any cause. Failure to achieve remission due to early death or non-response was considered as event at time zero. Survival was defined as the time of diagnosis to death from any cause or last follow-up. The Kaplan-Meier method was used to estimate survival rates, and differences were compared with the two-sided log rank test. Cox’s proportional hazards model was used for uni- and multivariate analyses. Cumulative incidence (CI) functions for competing events were constructed by the method of Kalbfleisch and Prentice, and were compared with the Gray’s test [36]. Results are presented as estimated probability of 5-year EFS (pEFS) and estimated cumulative incidence of relapse (pCIR) with standard error (± SE). Differences in the distribution of individual parameters among patient subsets were analyzed using Fisher’s exact test for categorized variables and the Mann-Whitney-U test for continuous variables. Logistical regression was used to analyze the effect of mutations on response variables (prednisone response, MRD). All statistical analyses were conducted using the SAS program (SAS-PC, v. 9.1, SAS Institute Inc.).

Experimental results were analyzed by Student’s t-tests as well as one-way and two-way ANOVA. If not stated otherwise in the figure legends, data are presented as average ± SE. Graphs and tables were prepared by using GraphPad Prism6, Microsoft Office 2010 (Excel, PowerPoint) and ImageJ 1.48v. Coffalyser software was used for MLPA analyses (available at http://www.mlpa.com).

## Results

### Deletions of *MAP3K7* are associated with *SIL-TAL1* fusions and with a mature T-ALL immunophenotype

**Fig. 2:**
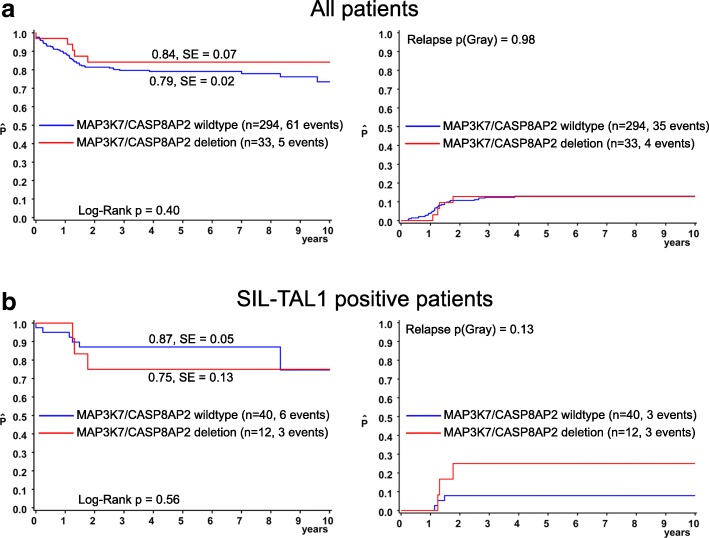
Deletion of *MAP3K7/CASP8AP2* does not affect outcome of pediatric T-ALL patients. Cumulative incidence of relapse (pCIR) and probability of event-free survival (pEFS) for T-ALL patients enrolled in ALL-BFM 2000 and ALL-BFM 2009 with *MAP3K7/CASP8AP2* wild type (blue) or *MAP3K7/CASP8AP2* deletion (red). Results are presented as estimated probability of 5-year EFS (pEFS) and estimated cumulative incidence of relapse (pCIR) with standard error (± SE). **a** T-ALL patients with or without *MAP3K7/CASP8AP2* deletion (*n* = 327). *MAP3K7/CASP8AP2* deletion neither affects the pEFS (p(Log-Rank) = 0.4) nor pCIR (p(Gray) = 0.98). **b** T-ALL patients with *SIL-TAL1* fusion (*n* = 52) who do or do not carry an additional *MAP3K7/CASP8AP2* deletion. *SIL-TAL1* positive patients harboring a deletion of *MAP3K7/CASP8AP2* show a trend towards a higher pCIR (p(Gray) = 0.13)

We performed MLPA on 327 primary T-ALL patient samples to detect deletions of 6q15 using probes directed against *MAP3K7* and *CASP8AP2*. All patients had been treated in the ALL-BFM 2000 or in the AIEOP-BFM ALL 2009 studies and clinical data were available (Table 1). All deletions of 6q15 were heterozygous. Thirty-two of 33 samples with a *MAP3K7* deletion also showed a loss of the adjacent *CASP8AP2* gene. We assume that all three genes located between *MAP3K7* and *CASP8AP2* (*GJA10, BACH2, MIR4464*) and a variable number of adjacent genes are co-deleted in these leukemias. Only in one sample a *MAP3K7* deletion without a *CASP8AP2* deletion was detected. These results support earlier results [4–9] showing that 6q15 deletions usually affect several genes. Analysis of clinical data of all 327 pediatric T-ALL patients who had been uniformly treated with ALL-BFM protocols revealed that the *MAP3K7/CASP8AP2* deletion is significantly associated with a mature T-ALL immunophenotype (*p* = 0.0005; Table 1), but not with any other clinical feature. Specifically, response to treatment as measured by prednisone response on day 8 and MRD assessment on days 33 and 78 after start of induction treatment did not differ between patients with or without a *MAP3K7* deletion (Table 1). There was no association of *MAP3K7/CASP8AP2* deletions with the cumulative incidence of relapse or overall survival (Fig. 2a). *MAP3K7/CASP8AP2* deletions were significantly more frequently observed in *SIL-TAL1* positive than in *SIL-TAL1* negative T-ALLs (*p* = 0.005). No other genetic feature including *NOTCH1* and *PTEN* mutations were associated with *MAP3K7* deletion. There was a trend for *SIL-TAL1* positive T-ALL patients harboring a *MAP3K7/CASP8AP2* deletion towards a higher risk of relapse compared to patients with *SIL-TAL1* fusion, but without *MAP3K7/CASP8AP2* deletion (p(Gray) = 0.13; Fig. 2b). Targeted sequencing identified a monoallelic point mutation in *MAP3K7* in less than 1% (1 of 147) of a subgroup of the T-ALL patients [37].

**Table 1:** Correlation of *MAP3K7/CASP8AP2* deletions with clinical features in primary T-ALL patients

Characteristic		*MAP3K7/CASP8AP2* wildtype (%)	*MAP3K7/CASP8AP2* deletion (%)	
All		294 (100)	33 (100)	
Sex	Male	213 (72.4)	27 (81.8)	*p* = 0.30
	Female	81 (27.6)	6 (18.2)	
Age	< 10	152 (51.7)	15 (45.5)	*p* = 0.58
	≥10	142 (48.3)	18 (54.5)	
WBC at diagnosis	< 10,000	26 (8.8)	2 (6.1)	*p* = 0.93
	10,000–50,000	69 (23.5)	7 (21.2)	
	50,000–100,000	55 (18.7)	7 (21.2)	
	≥100,000	144 (49.0)	17 (51.5)	
Mediastinal mass	Yes	155 (54.2)	16 (48.5)	*p* = 0.58
	No	131 (45.8)	17 (51.5)	
T-cell immunophenotype^a^	Early (Pro-/Pre-) T-ALL	89 (30.3)	7 (21.2)	*p* = 0.0005
	Cortical T-ALL	177 (60.2)	16 (48.5)	
	Mature T-ALL	24 (8.2)	10 (30.3)	
Prednisone response	PGR	173 (58.8)	17 (51.5)	*p* = 0.35
	PPR	113 (38.4)	16 (48.5)	
MRD on day 33	N/A	49 (16.7)	2 (6.1)	*p* = 0.18
	Negative	54 (18.4)	4 (12.1)	
	10^−4^	76 (25.9)	7 (21.2)	
	≥10^−3^	115 (39.1)	20 (60.6)	
MRD on day 78	N/A	42 (14.3)	2 (6.1)	*p* = 1.00
	< 10^−3^	221 (75.2)	27 (81.8)	
	≥10^−3^	31 (10.5)	4 (12.1)	
Risk Group 2000^b^	SR	41 (13.9)	3 (9.1)	*p* = 0.69
	MR	127 (43.2)	14 (42.4)	
	HR	126 (42.9)	16 (48.5)	
Genetics: *SIL-TAL1* fusion	Yes	39 (13.2)	12 (36.4)	*p* = 0.002
	No	255 (86.7)	21 (63.6)	

*WBC* White blood cell count, *PGR/PPR* Prednisone good/poor response, *MRD* Minimal residual disease, *SR/MR/HR* Standard/Medium/High risk. ^a^Early = Pro (cyCD3^+^, CD7^+^) and Pre (cyCD3^+^, CD2^+^ and/or CD5^+^ and/or CD8^+^); cortical (CD1a^+^); mature (CD1a^−^, sCD3^+^). cyCD3^+^: cytoplasmic CD3^+^; sCD3^+^: surface CD3^+^. ^b^The ALL-BFM 2000 overall risk classification defines three groups: SR - prednisone good response on day 8 (< 1000/μL leukemic blasts in peripheral blood) and complete cytomorphologic remission on day 33 and negative MRD on day 33 and day 78; MR - prednisone good response on day 8 and complete cytomorphologic remission on day 33 and MRD positive on day 33 and/or day 78, but < 10^−3^ on day 78; HR – prednisone poor response on day 8 or no complete cytomorphologic remission on day 33 or MRD on day 78 ≥ 10^− 3^ [3]

If numbers for clinical data sum up to less than 327, this feature was not available for all patients

### T-ALL cell lines can be efficiently transduced by adeno-associated viral vectors

To analyze the biological effects of a reduced *MAP3K7* expression, we chose three T-ALL cell lines (CCRF-CEM, Jurkat, MOLT-4) that do not carry a *MAP3K7* deletion as assessed by MLPA. *MAP3K7* mRNA expression levels were estimated by quantitative RT-PCR to be similar in all three cell lines and comparable to those in HEK293 cells (Additional file 1: Figure S1 A). We aimed at phenocopying a *MAP3K7* deletion by shRNA-mediated depletion. Because T-ALL cell lines are resistant to common methods of chemical transfection [38], we used an adeno-associated viral (AAV) vector-mediated transduction system to efficiently deliver shRNA to our cell lines. Flow cytometry analysis demonstrated transduction efficiencies mostly above 80% for the cell lines CCRF-CEM and Jurkat (Additional file 1: Figure S1 B). All subsequent experiments with these cell lines were performed after a transduction efficiency of at least 80% had been confirmed. However, transduction of MOLT-4 cells was less efficient and depended on the type of shRNA, so the threshold for further analysis in this cell line was set to a transduction efficiency of at least 60%. Depletion efficiency as assessed by quantitative RT-PCR and Western blotting was uniform, with both *MAP3K7* mRNA and MAP3K7 protein being in the range of 25% of that after treatment with non-silencing shRNA (Additional file 1: Figure S1C-D).

### MAP3K7 knockdown slows down proliferation in T-ALL cell lines

**Fig. 3:**
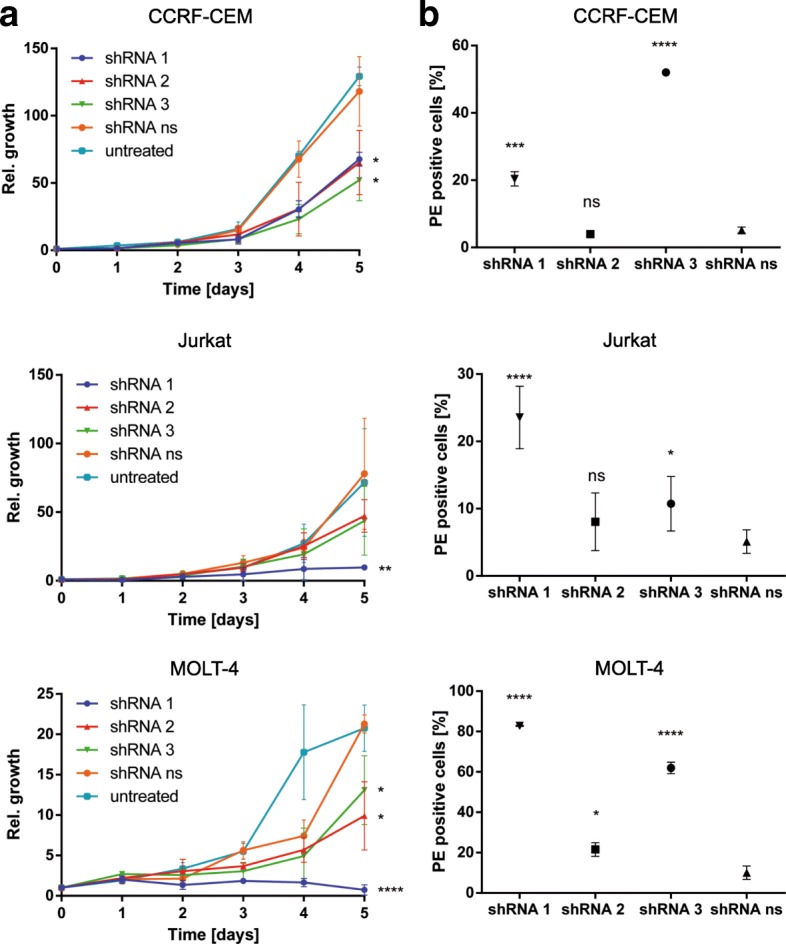
*MAP3K7* depletion decreases proliferation and induces apoptosis in T-ALL cell lines. T-ALL cell lines were transduced with AAV vectors coding for three different shRNAs (1, 2, 3) and one non-silencing shRNA (ns). Transduction efficiency was controlled by flow cytometry after 72 h of incubation. **a** *MAP3K7* depletion reduces proliferation of T-ALL cells. After exchanging the culture medium three days after transduction, cells were seeded at a density of 40 cells/μl. Every 24 h, an aliquot was stained by Trypan blue and vital cells were counted in a hemocytometer (Neubauer improved, Assistent). Relative proliferation was defined as the ratio of cell numbers on the day of interest over the starting cell number. Means of relative proliferation and standard deviations are given in the graph. Significance of differences in proliferation rates were calculated by two-way ANOVA and Student’s t-test compared to shRNA ns on day 5 of counting (* = *p* < 0.05, ** = *p* < 0.01, *** = *p* < 0.001, **** *p* < 0.0001, n(CCRF-CEM, MOLT-4) = 3, n(Jurkat) = 5). **b** *MAP3K7* depletion sensitizes T-ALL cells for apoptosis. Six days after transduction, apoptotic cells were stained with PE-conjugated Annexin V and measured by flow cytometry. Dot plot gates for untreated control were set to have less than 1% apoptotic cells. Gates of transduced cells were adjusted accordingly. The percentage of Annexin V-positive cells was compared between treatment with non-silencing shRNA (ns) and shRNAs 1, 2 and 3 directed against *MAP3K7*. Results are presented as means and standard deviations of PE-positive cells in percent. Significance was calculated by unpaired t-test compared to non-silencing shRNA (* = *p* < 0.05, ** = *p* < 0.01, *** = *p* < 0.001, **** *p* < 0.0001, n(CCRF-CEM, Jurkat) = 3, n(MOLT-4) = 6)

Treatment with anti-*MAP3K7* shRNA significantly slowed down proliferation in all three of the T-ALL cell lines analyzed (Fig. 3a). Five days after transduction, all three shRNAs resulted in a significant reduction of cell numbers compared to the non-silencing control (two-way ANOVA for treatment vs non-silencing control: p(CCRF-CEM) = 0.0044, p(Jurkat) = 0.0057, p(MOLT-4) = < 0.0001). Treatment with shRNA 1 in Jurkat and MOLT-4 cells completely abrogated cell proliferation and/or led to a loss of cells.

### MAP3K7 depletion induces apoptosis in T-ALL cell lines

In order to assess if decreasing cell numbers after depletion of *MAP3K7* were due to a higher rate of apoptosis, we performed an Annexin V assay six days after transduction. Treatment with anti-*MAP3K7* shRNA resulted in an increase in the proportion of Annexin V positive cells in all three cell lines of up to 8-fold (Fig. 3b). The extent of this effect depended on the cell type and the type of shRNA used. In all three cell lines, shRNA 2 had no or only a marginal effect on the proportion of Annexin V positive cells, possibly reflecting the relative low transduction efficiency for the corresponding shRNA construct. The strongest reduction of cell numbers was observed with the shRNA that most strongly induced apoptosis, suggesting that depletion of MAP3K7 impairs cell density by increasing the rate of apoptosis. However, the potential to induce apoptosis did not fully explain the effect on cell numbers, indicating that further mechanisms influence proliferation.

### Effects of MAP3K7 depletion are not mediated by NF-κB inactivation

**Fig. 4:**
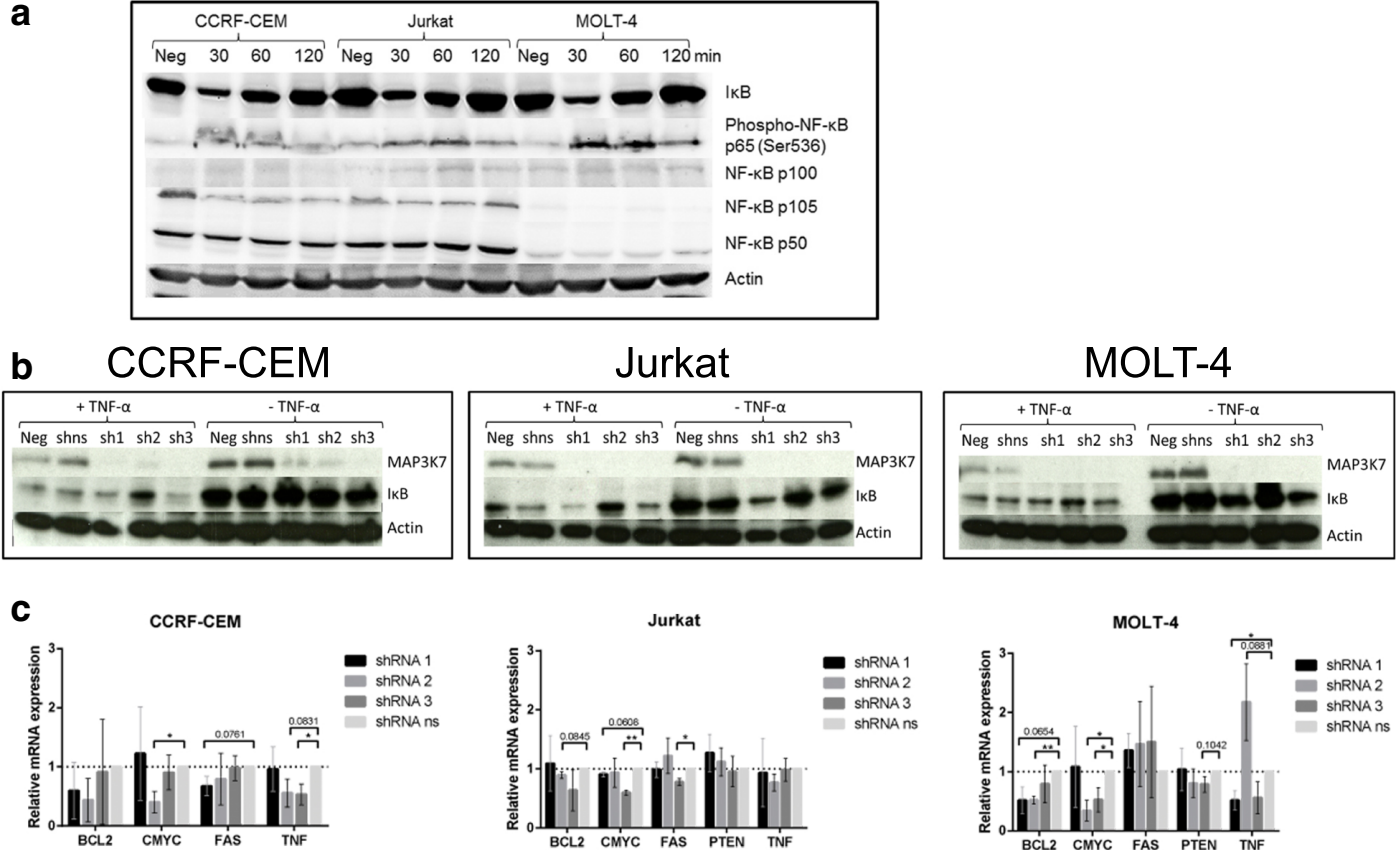
*MAP3K7* depletion does not inhibit NF-κB activation after stimulation of T-ALL cell lines with TNF-α. Six days after transduction with anti-*MAP3K7* shRNA, T-ALL cell lines were stimulated with TNF-α (10 ng/μl) for 30 min and whole cell lysates were analyzed by Western blotting. **a** Stimulation of non-transduced T-ALL cells results in degradation of IκB. **b** MAP3K7 depletion does not prevent degradation of IκB after stimulation with TNF-α. **c** Treatment with anti-*MAP3K7* shRNA did not consistently change expression patterns of NF-κB target genes in T-ALL cell lines. Six days after transduction with anti-*MAP3K7* shRNA, total RNA was extracted, cDNA was synthesized and *MAP3K7* mRNA expression quantified by qRT-PCR. HPRT1 was used as internal control. Mean values and SE of expression levels are given (*n* = 3). CCRF-CEM carries a *PTEN* deletion and does not express PTEN [62]

*MAP3K7* deficiency has been shown to inhibit NF-κB activation in different cell types [29, 32, 39]. To study the relevance of this interaction in T-ALL cells, we stimulated cell lines with TNF-α six days after transduction with shRNAs against *MAP3K7* and analyzed protein lysates by Western blotting for components of the NF-κB pathway (Fig. 4). Stimulation with TNF-α led to rapid and transient degradation of IκB and accumulation of phospho-NF-κB p65 (Ser536) in both untreated control cells and ns shRNA-treated cells, while other NF-κB proteins (p100, p105, p50) remained largely unaffected (Fig. 4a). Depletion of *MAP3K7* mediated by shRNAs 1 and 3 neither changed IκB levels in unstimulated cells nor did it prevent the degradation of IκB after stimulation with TNF-α. shRNA 2 partially impaired degradation of IκB (Fig. 4b).

Next, we asked if *MAP3K7* depletion influences expression levels of NF-κB target genes. We extracted total RNA from T-ALL cells after MAP3K7 depletion and analyzed mRNA expression levels of five NF-κB target genes (*BCL2* [40], *CMYC* [41], *PTEN* [42], *TNF*-α [43, 44], *FAS* [45, 46]) by quantitative RT-PCR. Expression of none of these genes was consistently changed by more than two-fold in any of the cell lines (Fig. 4c), indicating that the effect of NF-κB on gene expression does not depend on *MAP3K7*. We conclude that in T-ALL cell lines, *MAP3K7* is not required for the degradation of IκB and subsequent activation of NF-κB after stimulation with TNF-α.

## Discussion

We confirmed in a large cohort that heterozygous deletions of 6p15, including the *MAP3K7* locus, are a frequent event in T-ALL. In contrast, point mutations in *MAP3K7* were observed in less than 1% of primary T-ALLs [37]. *MAP3K7* deletions were associated with the presence of *SIL-TAL1* fusions and a mature immunophenotype. Although leukemias with TAL1 overexpression were described to reflect the late cortical stage of thymocyte differentiation [47], *SIL-TAL1* fusions were not associated with a certain immunophenotype in our and published cohorts [48]. We therefore hypothesize that the deletion of *MAP3K7/CASP8AP2* or another gene on 6q15 may directly influence the expression of T-cell surface markers. Alternatively, T-ALL cells that resemble a mature thymocyte may be less dependent on high expression levels of *MAP3K7* and thus less vulnerable to deletions of 6q15 in comparison to cortical or even less mature T-ALLs. No other clinical features were correlated with deletions of *MAP3K7*. Importantly, *MAP3K7* deletions neither predicted treatment response nor the risk of relapse. Previous studies identified several mostly overlapping regions on chr 6q that are recurrently deleted in pediatric T-ALL and T-lymphoblastic lymphoma (Fig. 1, [7]). The centromeric region is localized on 6q14 and 6q15 and involves, among others, *MAP3K7* and *CASP8AP2*. In a smaller cohort of patients, this deletion has been associated with a poor response to induction treatment [4]. However, we neither found an association of *MAP3K7/CASP8AP2* deletions with an unfavorable treatment response nor with the cumulative incidence of relapse. Obviously, deletions of *MAP3K7* are not a useful prognostic marker in the context of ALL-BFM 2000 and AIEOP-BFM ALL 2009 protocols. Future studies investigating the prognostic relevance of deletions on chr6q in T-ALL will ideally use high resolution mapping of copy number alterations in a large cohort of patients. Our work extends earlier findings that suggest that the effect of *MAP3K7* deletions on the prognosis of malignancies is highly dependent on the cell of origin: *MAP3K7* deletions are associated with advanced high-risk disease in prostate cancer [16, 27], but with a good prognosis in esophageal squamous cell carcinoma [49]. The effect on the prognosis of pediatric T-ALL seems to be very limited with current treatment protocols.

Transduction of T-ALL cell lines by AAV was efficient and resulted in transgene delivery efficiencies exceeding those typically reached by chemical transfection [38]. The simulation of *MAP3K7* deletion by AAV-mediated shRNA depletion resulted in slower proliferation and increased apoptosis in all cell lines analyzed. The specificity of the effect of MAP3K7 depletion was confirmed with three different shRNA constructs in three different cell lines. Of note, the restraints in insert size in our AAV vectors did not allow rescue experiments reversing the effects of the depletion by ectopic expression of *MAP3K7,* so we cannot fully exclude off target effects.

It has previously been shown that MAP3K7 is indispensable for thymocyte and T-cell development [24, 50]. Similar observations in different cancer models have primarily been attributed to an inactivation of the NF-κB pathway upon *MAP3K7* inhibition [25, 29]. For instance, chemical inhibition of MAP3K7 completely abolished NF-κB activation in AML cells, leading to cell death by apoptosis and decreased expression of IL8 [32]. The expression of a constitutively activated NF-κB p65 subunit only partially reduced these effects, indicating that *MAP3K7* signaling was not restricted to NF-κB activation [32]. Similarly, our results neither showed an effect on IκB levels nor on NF-κB target gene expression as a response to *MAP3K7* depletion. We conclude that the biologic effects of *MAP3K7* depletion were independent of the NF-κB pathway. Several genes recurrently mutated in T-ALL influence the MAP3K7/NF-κB signaling pathway. Most prominently, NOTCH1 directly interacts with NF-κB proteins and the IKK complex, leading to their activation [51]. Both MOLT-4 and CCRF-CEM cell lines carry *NOTCH1* mutations [52], and MAP3K7 might not be required additionally to activate NF-κB and the IKK complex. Furthermore, the function of *MAP3K7* may not only depend on mutations in genes that can activate or inactivate NF-κB, but also on the cell type and the maturation stage. Specifically, MAP3K7 has a critical role for NF-κB activation in naïve T-cells, but is dispensable in effector T-cells [53]. This observation may also explain why deletions of *MAP3K7* are found more frequently among leukemias with a more mature immunophenotype: Possibly “mature” lymphoblasts - in contrast to lymphoblasts resembling less mature precursors - do not depend on *MAP3K7* for the activation of the NF-κB pathway, which has been shown to be required for leukemogenesis in several models [51, 54–56]. As we did not find a direct effect of *MAP3K7* depletion on the activity of NF-κB, we suggest that *MAP3K7* has an effect on cell proliferation that is independent from NF-κB.

Notably, our data do not formally exclude that the low MAP3K7 protein levels remaining after AAV-mediated depletion have different biological effects than the higher MAP3K7 protein levels remaining in cells carrying heterozygous deletions. We never observed homozygous deletions of *MAP3K7* or the combination of a heterozygous deletion and a mutational inactivation of the remaining allele, indicating that some residual MAP3K7 activity is required for survival and proliferation of T-ALL cells. Although our results do not argue for a biological effect of monoallelic deletions of MAP3K7 in T-ALL, the fact that T-ALL cells require residual expression of *MAP3K7* may imply the potential of *MAP3K7* as a new treatment target: If *MAP3K7* is not a tumor suppressor but co-deleted with another, yet to be identified tumor suppressor on 6q15, it may behave as a “CYCLOPS” gene [57]. Accordingly, *MAP3K7* deletion may render leukemia cells highly vulnerable to inactivation of the remaining *MAP3K7* allele. Possible candidate drugs that may be able to exploit this potential Achilles’ heel are chemical MAP3K7 inhibitors like (5Z)-7-Oxozeaenol, LYTAK1, AZ-TAK1 and NG52, which showed anti-tumor effects in various in vivo and in vitro cancer models but do not appear suitable for the use in a clinical setting due to low selectivity [58]. However, the multikinase inhibitor sorafenib [59], which has already been proven to be clinically effective in the treatment of various malignancies, inhibits MAP3K7 and may specifically target leukemias with low expression of MAP3K7 [60, 61].

## Conclusions

Our results show that heterozygous *MAP3K7* deletions are recurrently found in T-ALL patients, but do not affect patients’ outcome in the context of ALL-BFM treatment protocols. On a cellular level, *MAP3K7* is essential for rapid proliferation and inhibition of apoptosis. In contrast to previous observations, we did not find inactivation of NF-κB to be the mechanism underlying the biological effects of *MAP3K7* inactivation in T-ALL cell lines. The complete absence of homozygous *MAP3K7* deletions and the proliferation arrest after efficient depletion indicate that *MAP3K7* may be indispensable for T-ALL cells and thus a potential target for treatment.

## Additional file

Additional file 1: Supplemental Methods: Details an patient data, AAV vector production, shRNA vector design. **Table S1.** Primers used for qRT-PCR. **Figure S1.** Transduction of T-ALL with anti-*MAP3K7* shRNA leads to an efficient knockdown. **Figure S2.** Plasmid map of pscAAV-CMV-GFP-U6-sh construct with anti-MAP3K7 shRNA. **Figure S3.** Transduction of T-ALL with anti-*MAP3K7* shRNA induces apoptosis. (ZIP 3146 kb)

## Abbreviations

AAV: Adeno-associated virus
GFP: Green fluorescent protein
MLPA: Multiplex ligation-dependent probe amplification
MOI: Multiplicity of infection
MRD: Minimal residual disease
PBS: Phosphate-buffered saline
pCIR: Estimated cumulative incidence of relapse
PCR: Polymerase chain reaction
PE: R-Phycoerythrin
pEFS: Probability of 5-year event free survival
PFA: Paraformaldehyde
qRT-PCR: Quantitative Real-Time PCR
RMD: Region of minimal deletion
shRNA: Short hairpin RNA
TAK1: TGF-beta activated kinase 1
T-ALL: T-cell acute lymphoblastic leukemia
TBS-T: Tris-buffered saline with 0.5% Tween-20
T-LBL: T-cell lymphoblastic lymphoma
