# Supplementary material for: MAP3K7 is recurrently deleted in pediatric T-lymphoblastic leukemia and affects cell proliferation independently of NF-κB
Source: BMC Cancer. 2018 Jun 18;18:663. doi: 10.1186/s12885-018-4525-0 (PMC6006985; doi:10.1186/s12885-018-4525-0)
Supplement: Supplementary file 1 — Supplemental Methods: Details an patient data, AAV vector production, shRNA vector design. Table S1. Primers used for qRT-PCR. Figure S1. Transduction of T-ALL with anti-MAP3K7 shRNA leads to an efficient knockdown. Figure S2. Plasmid map of pscAAV-CMV-GFP-U6-sh construct with anti-MAP3K7 shRNA. Figure S3. Transduction of T-ALL with anti-MAP3K7 shRNA induces apoptosis. (ZIP 3146 kb) [file 12885_2018_4525_MOESM1_ESM.zip › supplement_revisedR2.docx]

## Additional file 1

## Supplemental methods

**Detailed patient data information**

Patients who were treated for T-ALL either in the ALL-BFM 2000 trial or the AIEOP-BFM ALL 2009 trial and had given informed consent were included in the study if sufficient DNA from the time of diagnosis was available for genetic analysis. The ALL-BFM 200 study was approved by the institutional review board of the Hannover Medical School and other participating institutions, and the AIEOP-BFM ALL 2009 trial was approved by the institutional review board of the University of Schleswig-Holstein. Informed consent was obtained in accordance with the Declaration of Helsinki. The trials enrolled pediatric patients between 1 and 18 years of age. All T-ALL patient samples analyzed here were obtained from German treatment centers. Mononuclear cells were isolated from bone marrow (BM) samples and stored in liquid nitrogen or at -80°C until DNA extraction. All BM samples contained a blast percentage of 80% or more. Immunophenotyping was carried out as previously described [1], and the subclassification of T-ALL was performed according to the guidelines of the European Group for Immunological Characterization of Leukemias (EGIL) [2]. Early in vivo response to prednisone, defined as the cytoreduction to a 7-day prednisone treatment prophase and a single dose of intrathecal methotrexate on day 1, served to assess the effect of early treatment [3]. According to prednisone response, patients were classified into good responders (PGR: <1000 blasts/µL at day 8) or poor responders (PPR: ≥1000 blasts/µL at day 8). Treatment response was further defined by determination of minimal residual disease (MRD) kinetics that were assessed at two different time points, *i.e.*, at days 33 and 78 of treatment, respectively [4-7]. Allele-specific oligonucleotide-PCR protocols were used for quantitative detection of leukemic clone-specific immunoglobulin and T-cell receptor gene rearrangements as described previously [8, 9]. For treatment stratification, the ALL-BFM 2000 protocol distinguishes the standard risk group (MRD negative on days 33 and 78), the high-risk group (MRD ≥ 10^-3^ cells on day 78) and the intermediate-risk group (all others) [8]. Complete remission (CR) was defined as less than 5% blasts in the regenerating BM, the absence of leukemic blasts in the peripheral blood and cerebrospinal fluid, and no evidence of localized disease. Relapse was defined as recurrence of lymphoblasts or localized leukemic infiltrates at any site.

**AAV vector production**

AAV vector production was done as described previously [10]. For vector production, ten 145 mm dishes with 4 × 10^6^ HEK293T cells in 22 ml DMEM per dish were seeded two days before transfection. The cells were then triple-transfected with 14.6 µg each of adenoviral helper, AAV helper and AAV vector plasmid (numbers are for one dish) using polyethylenimine (PEI) as transfection reagent. Briefly, the DNA (43.8 μg in total) was diluted in distilled water to a volume of 790 µl. Directly before use, 790 µl of a NaCl solution (300 mM) was added and mixed by inverting. In another vial, 438 µl of distilled water and 790 µl of NaCl (300 mM) werde added to 352 µl of PEI (1 mg/ml in H_2_O) and mixed by inverting. PEI solution was added dropwise to DNA solution, vortexed briefly and incubated for at least 10 min at room temperature. The mixture was subsequently added dropwise to the medium (3.2 ml per dish) and incubated at 37°C for 3 days. The cells were then scraped into the medium and centrifuged at 400 g for 15 min, washed with 1× PBS and transferred to 50-ml falcon tubes. Next, the pellet was resuspended in 6 ml of lysis buffer (50 mM Tris-HCl, pH 8.5, 50 mM NaHCO_3_) and subjected to five freeze-thaw cycles (−80/37°C). The cell lysate was sonicated for 80 seconds (at 48 W) and incubated with 50 U benzonase per ml for 1 hour at 37°C, before cell debris was spun down at 400 g for 20 min at 4°C. For virus purification, the lysate was added to a preformed gradient of 15, 25, 40 and 60% iodixanol (OptiPrep in PBS-MK; 1× PBS with 1 mM MgCl_2_, 2.5 mM KCl) and centrifuged in a 70.1 Ti rotor (Beckman Coulter) at 50.000 rpm and 4°C for 2 h. Purified viruses were finally retrieved from the 40% iodixanol phase using needle and syringe, and stored in 50 µl of aliquots at −80°C. Virus titers were determined as vector genome copy numbers per ml via standard RT-PCR.

**shRNA vector design**

The basic double-stranded AAV vector pscAAV-CMV-GFP-U6-sh for shRNA cloning and expression has been reported [10]. The original vector contains an *egfp* gene under a CMV promoter and a *ccdB* gene under a U6 promoter, flanked by AAV-2 and AAV-4 ITR regions [11]. For cloning of shRNAs, oligonucleotides were designed that included an AAAA overhang on the 3’ end and a CACC overhang on the 5’ end, which are complementary to the overhangs resulting from digestion of the pscAAV-CMV-GFP-U6-sh plasmid with BbsI. The latter was performed to delete the *ccdB* gene, which would otherwise prevent growth of transformed bacteria. The shRNA-encoding oligonucleotides were annealed by heating to 95°C followed by slow cooling, and then ligated into the AAV vector plasmid (Suppl. Figure 1). The shRNA sequences are described within the main file. A corresponding plasmid containing a scrambled shRNA was used as non-silencing control.

Table S1. **Primer sequences for qRT-PCR.**

| Primer name | Sequence 5’ - 3’ |
| --- | --- |
| MAP3K7 FP | CCAACCTCAGAAAAGCCAC |
| MAP3K7 RP | GATAAGCCATTGGGATGGAG |
| PTEN FP | GGAAAGGGACGAACTGGTG |
| PTEN RP | CGGCTGAGGGAACTCAAAGT |
| C-MYC FP | GTCGTTTCCGCAACAAGTCCTCT |
| C-MYC RP | GATAAGCCATTGGGATGGAG |
| TNF-α FP | TGCACTTTGGAGTGATCGGC |
| TNF-α RP | GAGGAGGTTGACCTTGGTCTG |
| BCL2 FP | CTTTGAGTTCGGTGGGGTCA |
| BCL2 RP | GGGCCGTACAGTTCCACAAA |
| FAS FP | TGTGACCCTTGCACCAAATG |
| FAS RP | GAAGACAAAGCCACCCCAAG |
| HPRT1 FP | GACCAGTCAACAGGGGACAT |
| HPRT1 RP | AACACTTCGTGGGGTCCTTTTC |

FP = Forward primer, RP = Reverse primer.

## *Supplemental Figures*

**Supplemental Figure 1
Transduction of T-ALL with anti-*MAP3K7* shRNA leads to an efficient knockdown.** T-ALL cells were seeded at a density of 40 cells/µl in 12-well plates and infected with the anti-*MAP3K7* shRNA-encoding AAV vectors at a MOI between 1*10^4^ to 5*10^5^.
(A) Relative mRNA expression of *MAP3K7* was detected in T-ALL cell lines and HEK293T. Total RNA was extracted from the T-ALL cell lines and the HEK293T cells and cDNA was synthesized. *MAP3K7* and *HPRT1* mRNA expression was measured by qRT-PCR. The housekeeping gene *HPRT1* was used for a comparison of *MAP3K7* CT (cycle threshold) values (n=3). Corrected CT values were normalized against the expression of HPRT1.
(B) AAV effectively transduces T-ALL cell lines. T-ALL cell lines were transduced and incubated with AAV for 72 hours. Transduction rates were measured by flow cytometry. Box and whiskers plots are shown with the range of minimum to maximum value. Measured data points are given in background (n=10-16).
(C) Anti-*MAP3K7* shRNA leads to an efficient knockdown of *MAP3K7* mRNA in T-ALL cell lines. Six days after transduction, total RNA was extracted, cDNA synthesized and *MAP3K7* mRNA expression measured by qRT-PCR. *HPRT1* was used for internal control. Mean values and SE of expression levels are given. The significance of the decrease in mRNA levels was analysed by unpaired t-test in comparison with shRNA ns constructs (with Welch’s correction due to unequal variance; *p<0.05, **p<0.01, ***p<0.001, ****p<0.00001, n=4).
(D) Representative Western blots and quantification results illustrating *MAP3K7* protein knockdown in T-ALL cell lines. Six days after transduction, whole cell lysates were extracted. For each cell line, one representative Western blot is shown. Western blots were quantitatively analyzed by the use of ImageJ [12]. Mean values and SE of protein expression levels are given. Significance of anti-*MAP3K7* treatment on protein expression levels was calculated by one-way ANOVA (n(shRNA ns, 1) = 11, n(shRNA 2, 3) = 7).

**Supplemental Figure 2
Plasmid map of pscAAV-CMV-GFP-U6-sh construct with anti-MAP3K7 shRNA.**

**Supplemental Figure 3
Transduction of T-ALL with anti-*MAP3K7* shRNA induces apoptosis.** T-ALL cell lines were transduced with AAV vectors coding for three different shRNAs (1, 2, 3) and one non-silencing shRNA (ns). Transduction efficiency was controlled by flow cytometry after 72 hours of incubation. Six days after transduction, apoptotic cells were stained with PE-conjugated Annexin V. Flow cytometry measured PE signal intensity (y-axis) vs. forward scatter (x-axis). Dot plot gates for untreated control were set to have less than 1% apoptotic cells. Gates of transduced cells were adjusted accordingly. For simplicity dot blots of single conditions were merged into one combined figure.

**References**

1. Ludwig WD, Rieder H, Bartram CR, Heinze B, Schwartz S, Gassmann W, Loffler H, Hossfeld D, Heil G, Handt S *et al*: **Immunophenotypic and genotypic features, clinical characteristics, and treatment outcome of adult pro-B acute lymphoblastic leukemia: results of the German multicenter trials GMALL 03/87 and 04/89**. *Blood* 1998, **92**(6):1898-1909.

2. Bene MC, Castoldi G, Knapp W, Ludwig WD, Matutes E, Orfao A, van't Veer MB: **Proposals for the immunological classification of acute leukemias. European Group for the Immunological Characterization of Leukemias (EGIL)**. *Leukemia* 1995, **9**(10):1783-1786.

3. Riehm H, Reiter A, Schrappe M, Berthold F, Dopfer R, Gerein V, Ludwig R, Ritter J, Stollmann B, Henze G: **[Corticosteroid-dependent reduction of leukocyte count in blood as a prognostic factor in acute lymphoblastic leukemia in childhood (therapy study ALL-BFM 83)]**. *Klin Padiatr* 1987, **199**(3):151-160.

4. Flohr T, Schrauder A, Cazzaniga G, Panzer-Grumayer R, van der Velden V, Fischer S, Stanulla M, Basso G, Niggli FK, Schafer BW *et al*: **Minimal residual disease-directed risk stratification using real-time quantitative PCR analysis of immunoglobulin and T-cell receptor gene rearrangements in the international multicenter trial AIEOP-BFM ALL 2000 for childhood acute lymphoblastic leukemia**. *Leukemia* 2008, **22**(4):771-782.

5. Hansen-Hagge TE, Yokota S, Bartram CR: **Detection of minimal residual disease in acute lymphoblastic leukemia by in vitro amplification of rearranged T-cell receptor delta chain sequences**. *Blood* 1989, **74**(5):1762-1767.

6. van Dongen JJ, Seriu T, Panzer-Grumayer ER, Biondi A, Pongers-Willemse MJ, Corral L, Stolz F, Schrappe M, Masera G, Kamps WA *et al*: **Prognostic value of minimal residual disease in acute lymphoblastic leukaemia in childhood**. *Lancet* 1998, **352**(9142):1731-1738.

7. Willemse MJ, Seriu T, Hettinger K, d'Aniello E, Hop WC, Panzer-Grumayer ER, Biondi A, Schrappe M, Kamps WA, Masera G *et al*: **Detection of minimal residual disease identifies differences in treatment response between T-ALL and precursor B-ALL**. *Blood* 2002, **99**(12):4386-4393.

8. Schrappe M, Valsecchi MG, Bartram CR, Schrauder A, Panzer-Grumayer R, Moricke A, Parasole R, Zimmermann M, Dworzak M, Buldini B *et al*: **Late MRD response determines relapse risk overall and in subsets of childhood T-cell ALL: results of the AIEOP-BFM-ALL 2000 study**. *Blood* 2011, **118**(8):2077-2084.

9. Koehler R, Bartram CR: **[Molecular genetic detection of minimal residual disease (MRD) in children with acute lymphoblastic leukemia]**. *Klin Padiatr* 2013, **225 Suppl 1**:S40-44.

10. Borner K, Niopek D, Cotugno G, Kaldenbach M, Pankert T, Willemsen J, Zhang X, Schurmann N, Mockenhaupt S, Serva A *et al*: **Robust RNAi enhancement via human Argonaute-2 overexpression from plasmids, viral vectors and cell lines**. *Nucleic Acids Res* 2013, **41**(21):e199.

11. Grimm D, Streetz KL, Jopling CL, Storm TA, Pandey K, Davis CR, Marion P, Salazar F, Kay MA: **Fatality in mice due to oversaturation of cellular microRNA/short hairpin RNA pathways**. *Nature* 2006, **441**(7092):537-541.

12. Schneider CA, Rasband WS, Eliceiri KW: **NIH Image to ImageJ: 25 years of image analysis**. *Nat Methods* 2012, **9**(7):671-675.
