# Supplementary figures and images for: MAP3K7 is recurrently deleted in pediatric T-lymphoblastic leukemia and affects cell proliferation independently of NF-κB
Source: BMC Cancer. 2018 Jun 18;18:663. doi: 10.1186/s12885-018-4525-0 (PMC6006985; doi:10.1186/s12885-018-4525-0)

**A**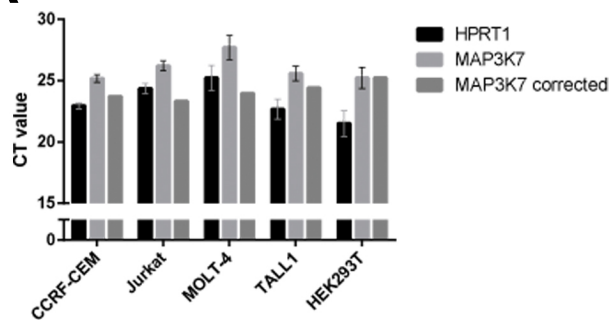**B**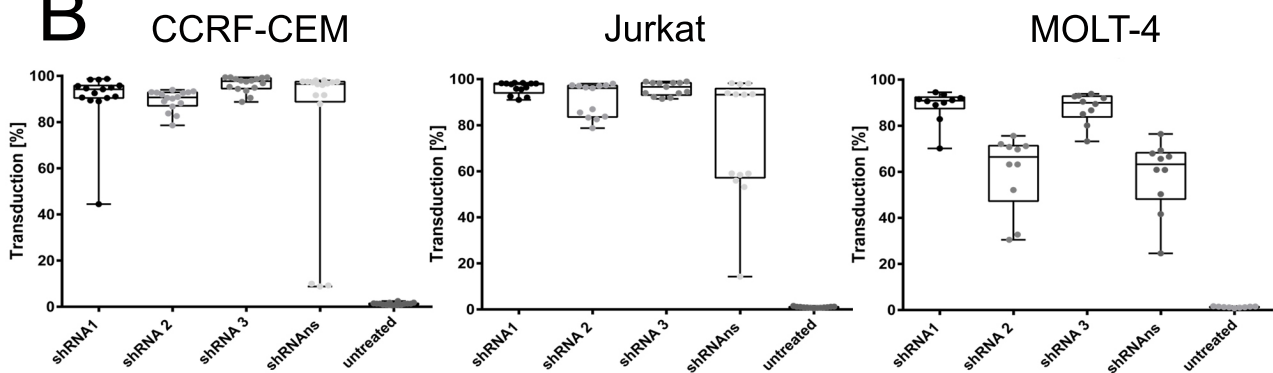**C**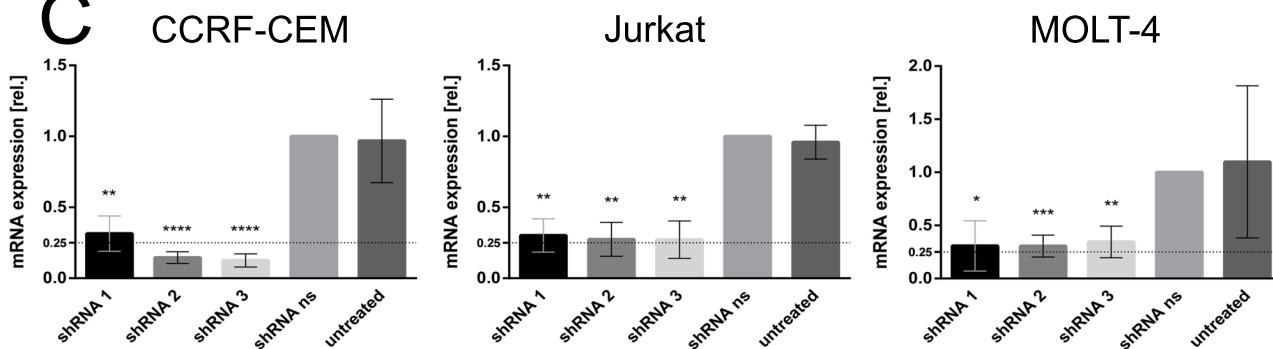**D**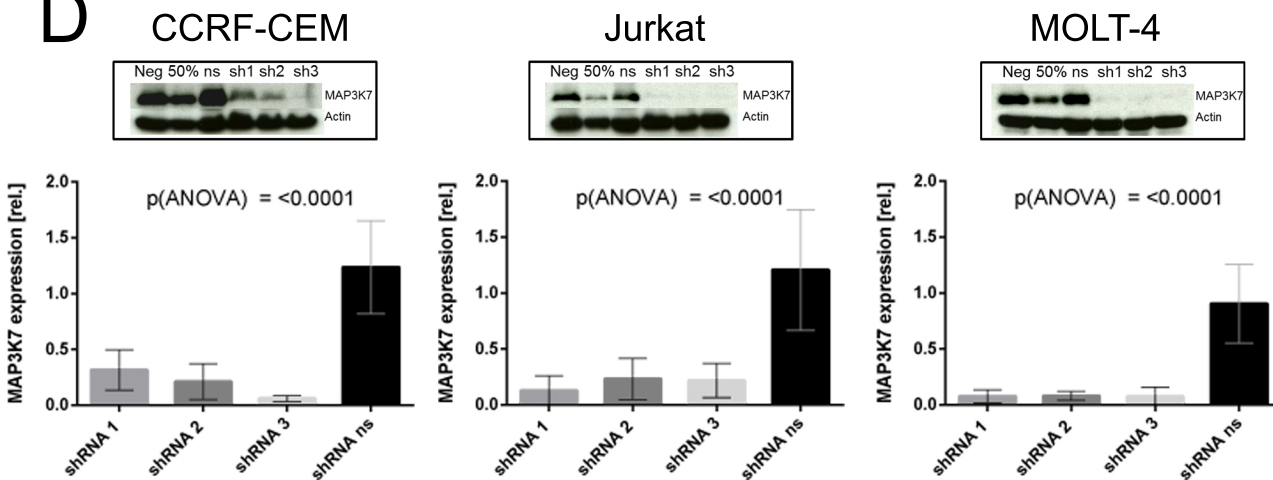

Supplement: Supplementary file 1 — Supplemental Methods: Details an patient data, AAV vector production, shRNA vector design. Table S1. Primers used for qRT-PCR. Figure S1. Transduction of T-ALL with anti-MAP3K7 shRNA leads to an efficient knockdown. Figure S2. Plasmid map of pscAAV-CMV-GFP-U6-sh construct with anti-MAP3K7 shRNA. Figure S3. Transduction of T-ALL with anti-MAP3K7 shRNA induces apoptosis. (ZIP 3146 kb) [file 12885_2018_4525_MOESM1_ESM.zip › Fig_S2R2.pdf]

# CCRF-CEM

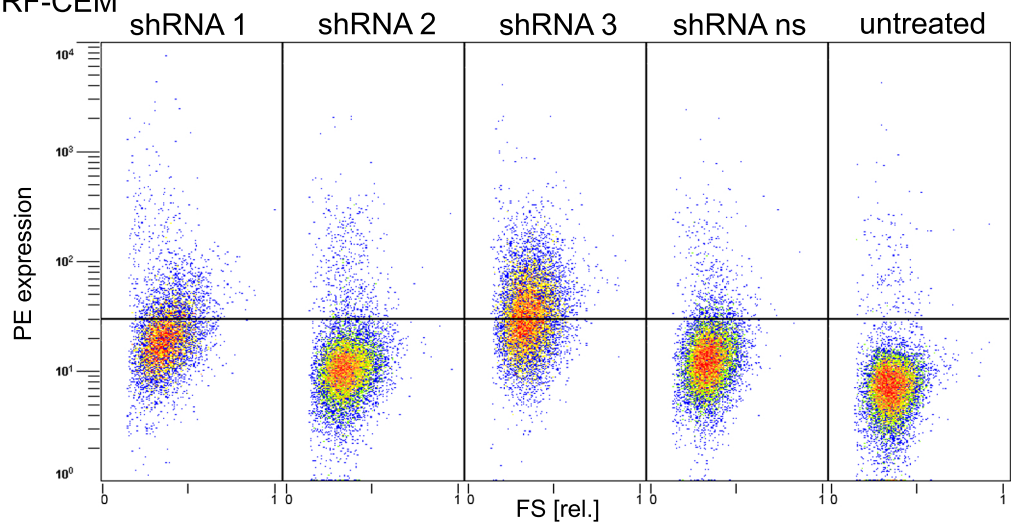

# Jurkat

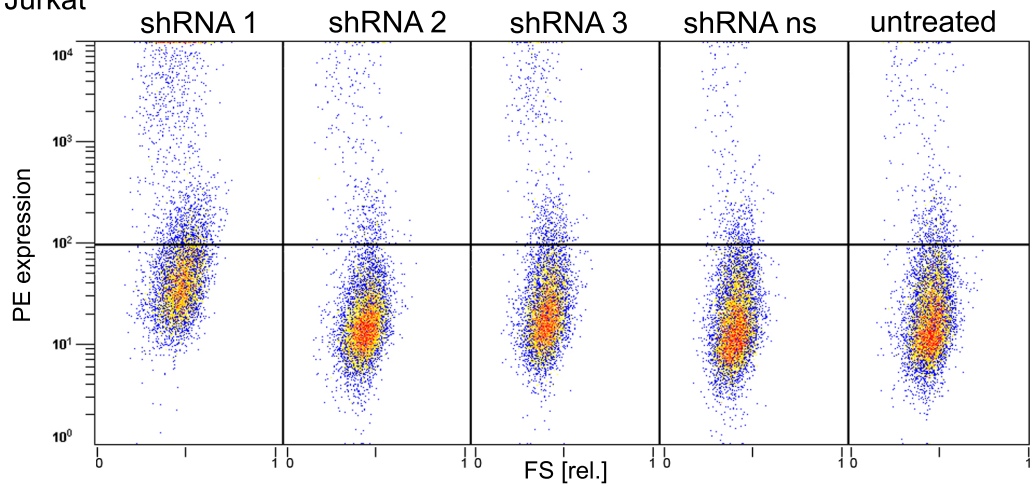

# MOLT-4

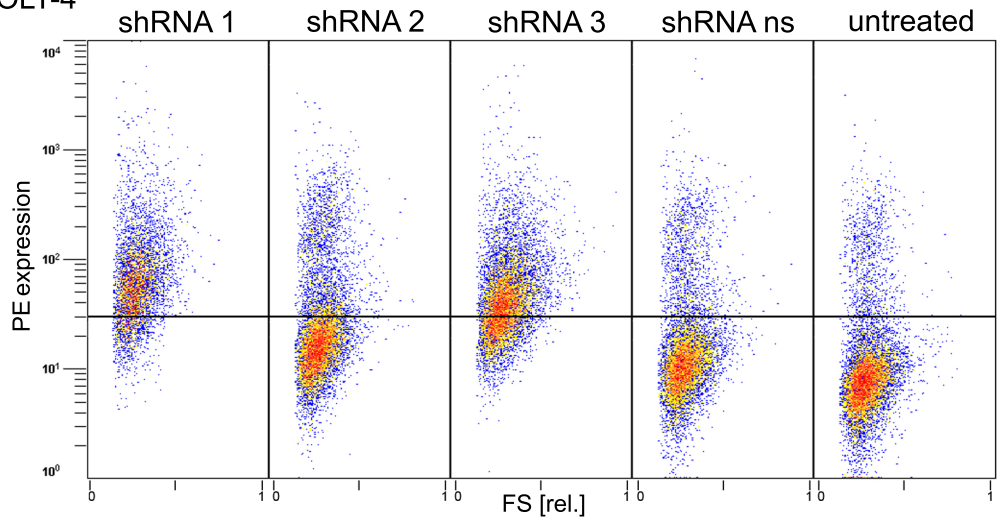

Supplement: Supplementary file 1 — Supplemental Methods: Details an patient data, AAV vector production, shRNA vector design. Table S1. Primers used for qRT-PCR. Figure S1. Transduction of T-ALL with anti-MAP3K7 shRNA leads to an efficient knockdown. Figure S2. Plasmid map of pscAAV-CMV-GFP-U6-sh construct with anti-MAP3K7 shRNA. Figure S3. Transduction of T-ALL with anti-MAP3K7 shRNA induces apoptosis. (ZIP 3146 kb) [file 12885_2018_4525_MOESM1_ESM.zip › Suppl. Figure 3R2.pdf]

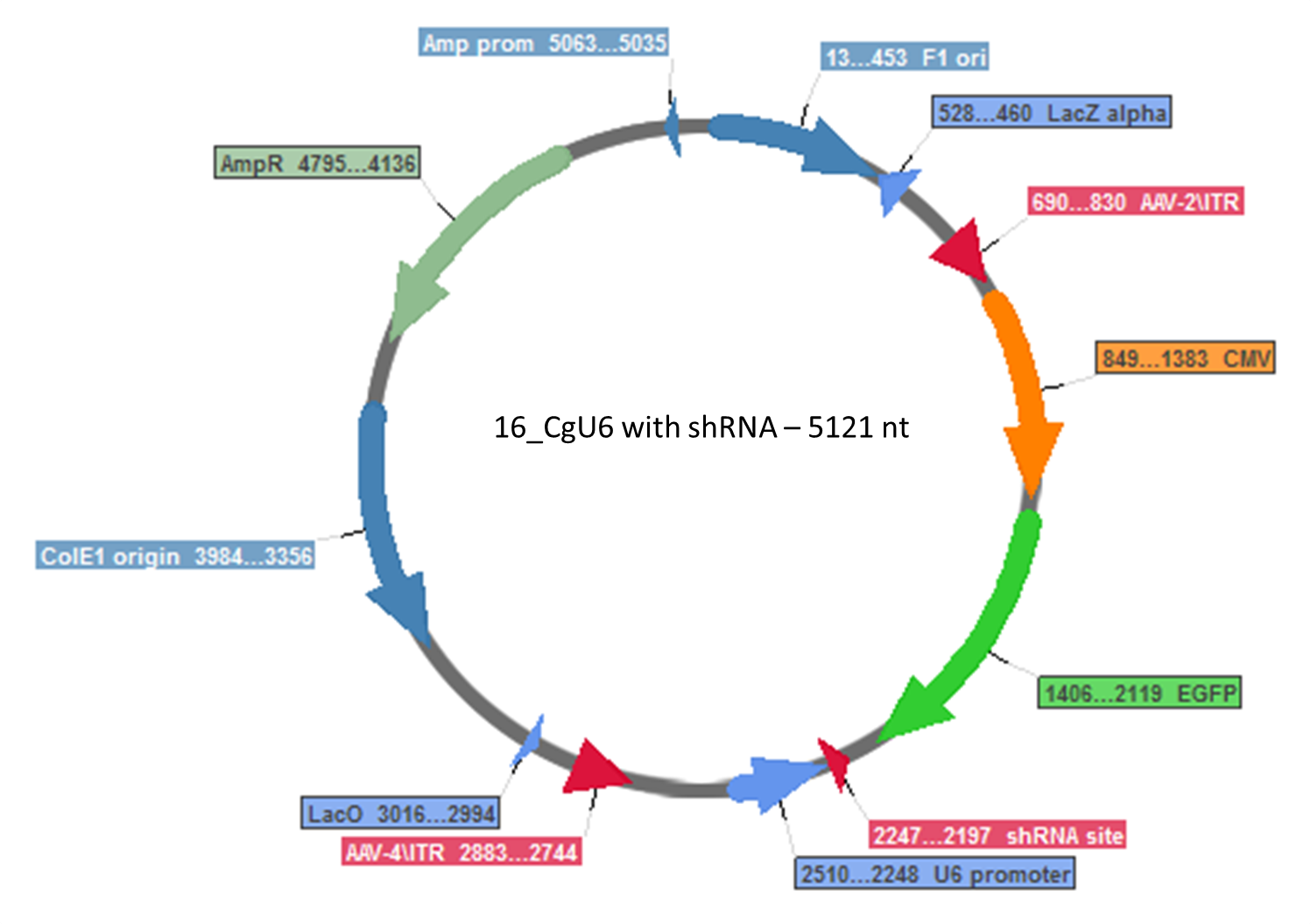

Supplement: Supplementary file 1 — Supplemental Methods: Details an patient data, AAV vector production, shRNA vector design. Table S1. Primers used for qRT-PCR. Figure S1. Transduction of T-ALL with anti-MAP3K7 shRNA leads to an efficient knockdown. Figure S2. Plasmid map of pscAAV-CMV-GFP-U6-sh construct with anti-MAP3K7 shRNA. Figure S3. Transduction of T-ALL with anti-MAP3K7 shRNA induces apoptosis. (ZIP 3146 kb) [file 12885_2018_4525_MOESM1_ESM.zip › Fig_S1_vectormapR2.png]
